# VIRTUAL COMPARATIVE GLOBAL AGING CURRICULUM/COURSE – STUDENT GLOBAL COMPETENCES QUALITATIVE THEMATIC ANALYSIS

**DOI:** 10.1093/geroni/igae098.3927

**Published:** 2024-12-31

**Authors:** Alina Zurek, Grzegorz Zurek, Lyn Holley

**Affiliations:** University of Wroclaw, Wroclaw, Dolnoslaskie, Poland; Wroclaw University of Health and Sport Sciences, Wroclaw, Dolnoslaskie, Poland; University of Nebraska at Omaha, Omaha, Nebraska, United States

## Abstract

Presents global competences acquired by students of the international virtual course Comparative Global Aging implemented in the years 2018-2023. The course is the result of American-Polish cooperation between the University of Nebraska in Omaha and two Polish universities - the University of Wrocaw and the University School of Physical Education in Wrocław. The content of thirty-seven Learning Journals of students from 10 European countries studying in Poland in the ERASMUS student exchange program and UNO students was subjected to qualitative analysis using MAXQDA software. Discuss the results in the three distinguished dimensions of global competences - cognitive, socio-emotional and ethical, and five thematic clusters: Critical and Constructive Thinking, Self-Awareness and Self-Direction in Education, Effective Cross-Cultural Cooperation, Open Intergenerational Communication, Innovation in Action and Problem Solving. Learning outcomes achieved by students, i.e. the ability to examine local, global and cross-cultural issues in the topic of ageing, understand and appreciate the perspectives of people living in other countries and cultures, engage in open and effective interactions, and work to solve the problems of older people using a broad global perspective of ageing countries and societies

